# Neuropsychiatric symptoms in Multiple Sclerosis (MS): Case Report of a First Manic Episode in a Patient with Suspected MS

**DOI:** 10.1192/j.eurpsy.2024.895

**Published:** 2024-08-27

**Authors:** M. A. N. C. Pais, T. M. F. Fernandes, M. Cagigal

**Affiliations:** ^1^Department of Psychiatry, Centro Hospitalar e Universitário de Coimbra, Coimbra; ^2^Department of Psychiatry, Centro Hospitalar e Universitário de Coimbra, Coimba, Portugal

## Abstract

**Introduction:**

Multiple Sclerosis (MS) is an inflammatory disease affecting primarily the central nervous system, characterized by focal lesions of white-matter demyelination. It can present with a variety of neurological symptoms, including monocular vision loss, sensory loss, paresthesias, limb weakness, ataxia and bladder dysfunction, and has a typically chronic and progressive course. Neuropsychiatric manifestations including depressive or manic symptoms, anxiety disorders and psychosis, are also frequently observed, and are of particular importance to mental health practitioners.

**Objectives:**

To describe a case of a 45-year-old female patient with a history of suspected MS presenting with manic symptoms, and to discuss the possible neuropsychiatric manifestations of Multiple Sclerosis.

**Methods:**

Clinical case report and literature review.

**Results:**

A 45-year-old woman was brought to the emergency department presenting with severe acute agitation, irritable mood, rapid speech and persecutory delusions. She had no prior history of neuropsychiatric symptoms, but her medical history was notable for a suspected diagnosis of MS, having suffered an episode of optic neuritis 16 years before the present episode. Magnetic Ressonance Imaging performed 3 months before emergency admission documented non-specific white-matter lesions presenting as hyper-intense in long TR sequences, as well as a cervical lesion of atypical characteristics, representing possible spondylotic myelopathy or demyelination. A head CT performed at emergency admission did not reveal relevant acute findings. The patient was hospitalized and initiated risperidone and valproic acid therapy. She responded favorably to medication, with progressive stabilization of mood and remission of delusional ideas over three weeks.

**Conclusions:**

Neuropsychiatric symptoms are a common and concerning manifestation of Multiple Sclerosis. The present case illustrates that clinicians should be on alert for signs of mood and psychotic symptoms in patients with suspected or confirmed MS, as these can manifest at any point during the disease course.

**Disclosure of Interest:**

None Declared

